# The Use of an Intraoral Scanner for the Fabrication of Maxillary Obturator Prosthesis in a Young Adult With Oronasal Fistula: A Case Report

**DOI:** 10.1155/crid/1167521

**Published:** 2025-07-26

**Authors:** Sayed Shojaedin Shayegh, Zahra Mohammadi, Mohammad Amin Bafandeh, Mohammad Reza Nazemalroaya

**Affiliations:** ^1^ Department of Prosthodontics, Faculty of Dentistry, Shahed University, Tehran, Iran, shahed.ac.ir

**Keywords:** 3D printing, cleft palate, digital approach, digital impression, prosthesis

## Abstract

**Background:**

Cleft lip and palate require complex treatment, often involving early surgery. Postoperative complications, such as palatal fistulas, can impair speech and feeding. While surgical correction is standard, large fistulas may pose challenges due to age, cost, and recurrence risks. Obturator prostheses provide a nonsurgical alternative, but digital impression techniques for their fabrication are underutilized. This case report explores intraoral digital impressions for creating obturator/speech aid appliances in a patient with cleft palate deformities.

**Methods:**

A 17‐year‐old female with Class III malocclusion on a Class III skeletal base and increased facial proportion and IOFTN score of 5, with cleft palate‐related eating difficulties, missing anterior teeth, and worn dentition, underwent intraoral scanning. Digital files were used to fabricate a premolar‐to‐premolar obturator, with relief areas and teeth arranged on printed casts.

**Results:**

The appliance effectively addressed functional and aesthetic concerns.

**Conclusion:**

Digital impressions offer precise, efficient, and comfortable fabrication of obturator prostheses compared to conventional methods. Despite initial costs, they reduce chair time, enhance accuracy for dental hard tissues, and improve patient experience, particularly for young patients with cleft lip and palate.

## 1. Introduction

Patients born with cleft lip and palate often require a comprehensive treatment approach encompassing both physical and psychosocial interventions to ensure a quality life [1]. Typically, primary surgical repair of the cleft occurs early in life, often before the age of 2, to promote normal development of essential functions such as speech, hearing, and swallowing [2, 3].

However, despite early intervention, complications such as palatal fistulas may arise following palatoplasty, necessitating additional surgical correction to mitigate their adverse effects on speech and feeding [3, 4]. The reported incidence of these fistulas varies widely, reflecting inconsistencies in definitions, classification systems, and reporting periods across different studies [5–7].

The primary approach to repairing palatal fistulas is through surgery, yet for large oronasal fistulas (L‐ONFs), this option may not be feasible for certain patients, influenced by factors like age and cost [8, 9]. Additionally, the recurrence rate following ONF repair surgery is considerable, spanning from 0% to 43% [2, 10, 11]. Given the challenges posed by surgical constraints and the elevated risks of morbidity and ONF recurrence, the use of obturators emerges as an appealing alternative. Obturator prostheses offer a nonsurgical alternative, effectively sealing the communication between the oral and nasal cavities to facilitate speech development and prevent food passage [3, 12, 13]. These appliances also play a crucial role in ensuring proper nutrition by reducing nasal regurgitation and feeding time [14].

In recent years, digital impression techniques have emerged as a promising alternative to conventional methods for obtaining accurate impressions of dental arches, offering enhanced patient comfort and cost‐effectiveness [15, 16]. Digital impressions have been shown to be as precise as analog impressions and can potentially reduce time and costs associated with the fabrication process [17, 18].

However, the adoption of digital technology in the fabrication of obturators and speech aid devices for children with cleft lip and palate deformities remains limited, primarily due to initial financial investments, operator training requirements, and ongoing maintenance costs [19]. Despite these challenges, digital impression techniques hold promise for improving the efficiency and accuracy of treatment protocols for this patient population.

In light of these considerations, the presented case report aims to showcase the clinical application of intraoral digital impressions in fabricating obturator/speech aid appliances for a patient with cleft palate deformities, highlighting the potential benefits of integrating digital technology into current treatment practices.

## 2. Clinical Report

**Figure 2:**
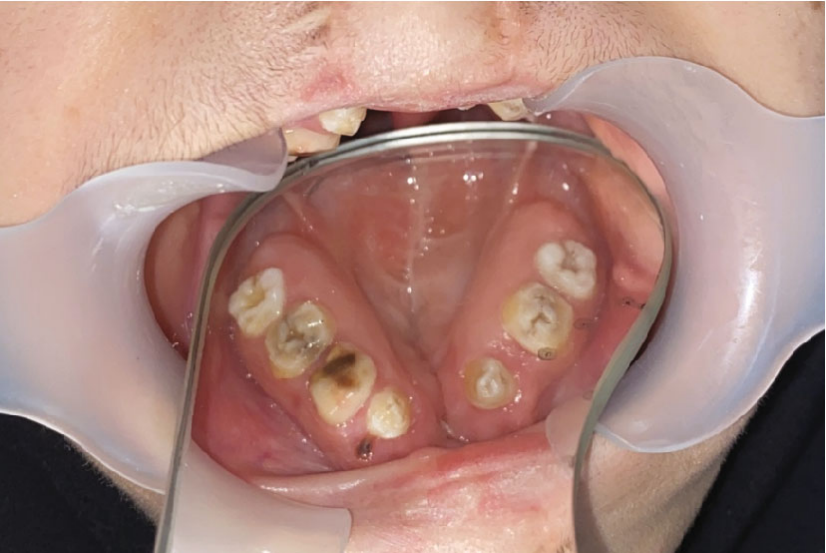
Mirror view of the maxillary arch. Notice the very short clinical crowns and lack of enamel. The oronasal fistula is on the hard palate between two canines, with a size of about 4.5 × 7.5 mm.

In October 2023, a 17‐year‐old female patient sought evaluation at the Prosthodontics Department of the University’s Dental School following unsuccessful attempts to secure treatment elsewhere. She presented with functional challenges in mastication and significant psychological distress resulting from the absence of her anterior dentition, which adversely affected her confidence and aesthetic self‐perception. The patient exhibited a normal maximum mouth opening, without indications of temporomandibular joint (TMJ) dysfunction or any reported history of parafunctional habits. Visual documentation, as depicted in Figure 1a, has been obtained with explicit patient consent. This patient has a history of bilateral cleft lip and palate, presenting with Class III malocclusion on a Class III skeletal base and increased facial proportions and IOFTN score of 5 [20] complicated by missing teeth (12, 13, 22, 23, and 25) and hypoplastic dentition characterized by pits and linear forms. Examinations revealed a 4.5 × 7.5 mm cavity on the palate, resulting in impacted anterior teeth. Furthermore, other affected teeth displayed enamel deficiencies and notably short anatomical clinical crowns, as depicted in Figure 2. The absence of adequate retention from the existing dentition appeared to have hindered the fabrication of both a prosthetic obturator and a cosmetic prosthesis up to this point.

Figure 1(a) Photograph of the patient from the first visit. (b) The patient after receiving the prosthesis.

**Figure:**
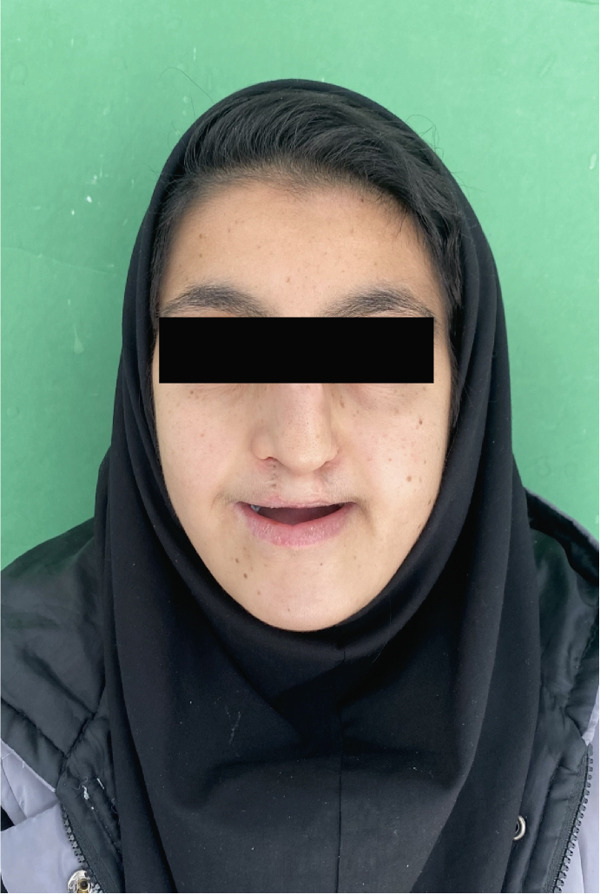
(a)

**Figure:**
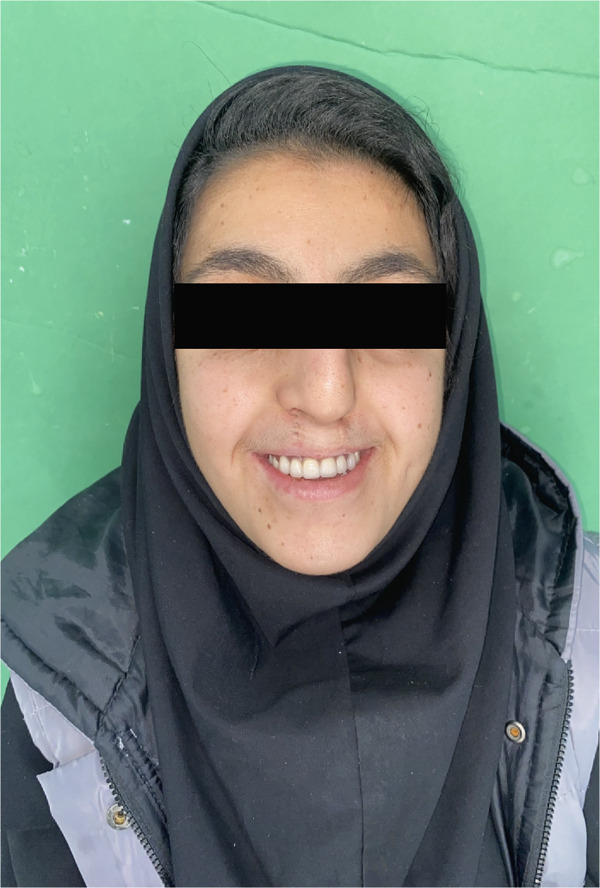
(b)

In patients with cleft lip and palate, various treatment options are available, including fixed partial dentures, implant‐supported prostheses, orthodontics, orthognathic surgery, and obturators. In this article, an obturator was utilized. The choice of this treatment was based on the patient’s reluctance to pursue implants and orthognathic surgery due to financial constraints and concerns about the invasiveness of surgery. Additionally, there was insufficient bone in the area to perform implant surgery, and after consulting with orthodontic specialists, it was determined that resolving the patient’s issue with orthodontics alone without additional surgery was not feasible. Since the patient was still growing and her supporting teeth had short crown heights, and because the soft tissue defect in the area could not be reconstructed with this type of prosthesis, this option was not selected either. The premolar‐to‐premolar obturator is an immediate solution that provides sufficient aesthetic appeal and adequate retention stability, while also being a noninvasive option.

**Figure 4:**
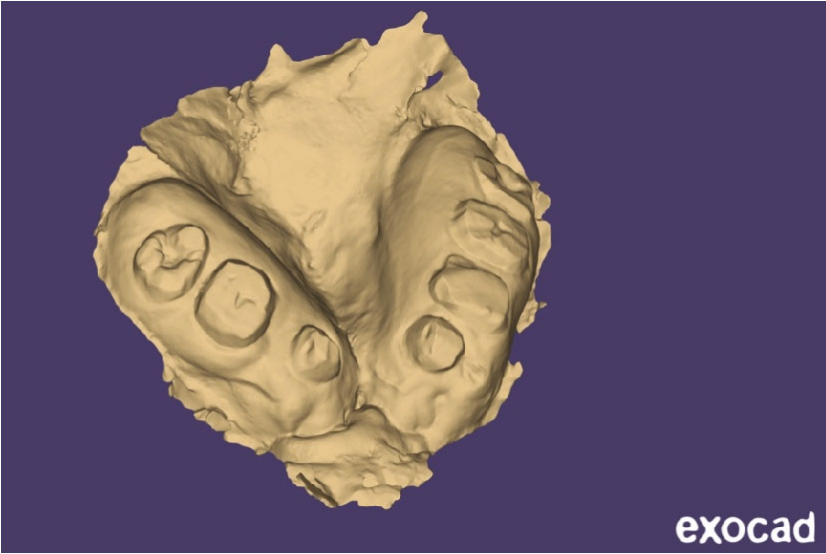
STL file of the maxillary arch in exocad.

Instead of going with conventional impressions, intraoral scanning utilizing an iAtonS 88dent scanner was conducted (Figure 3). The scanner was calibrated, and the software was updated according to the manufacturer’s instructions. Scanning was started from the right maxillary quadrant, and the scanning strategy involved first scanning the occlusal surface, then the buccal surface, and finally, the palatal surface and cleft area. Subsequently, the acquired STL and PLY files were sent to the laboratory (Figure 4), enabling the fabrication of jaw cast prints using a Phrozen 3D printer (Figure 5).

Figure 3Scanning with intraoral scanner: (a) the maxillary arch and (b) in occlusion.

**Figure:**
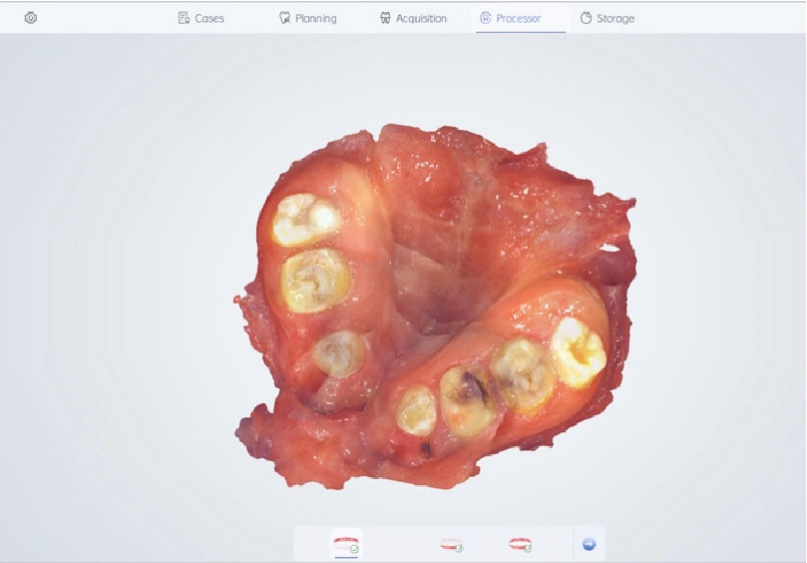
(a)

**Figure:**
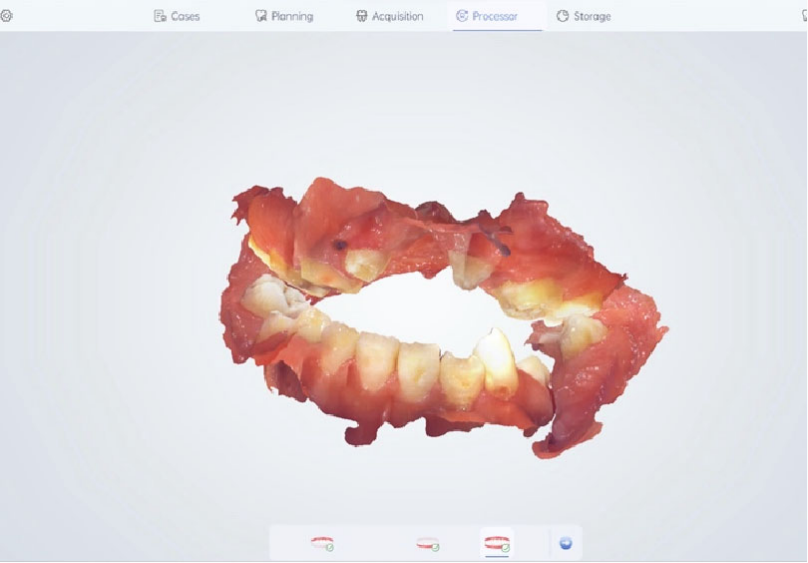
(b)

Figure 53D printed cast. (a) Mandibular arch and (b) maxillary arch.

**Figure:**
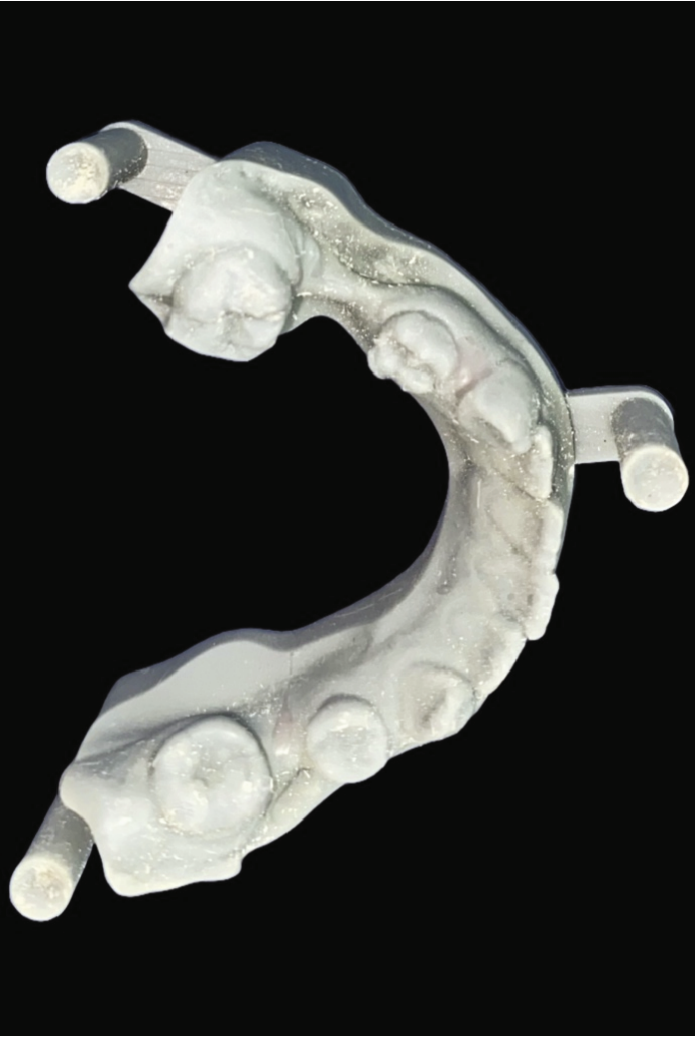
(a)

**Figure:**
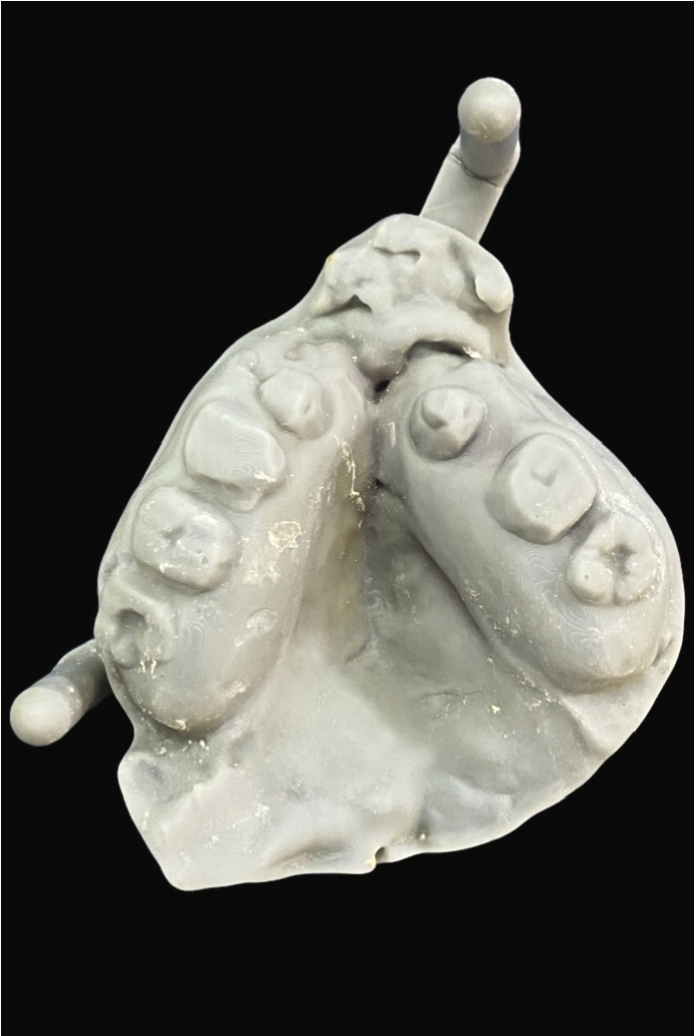
(b)

**Figure 6:**
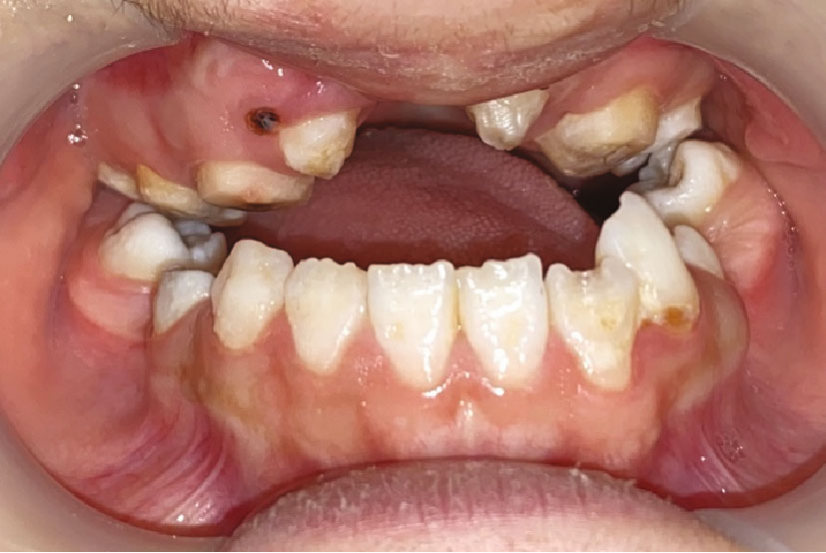
View of the patient’s mouth in occlusion. Notice that only the second molars are in occlusion.

The proposed treatment plan for the patient involved creating an obturator spanning from the right second premolar to the left second premolar to address both masticatory and aesthetic concerns simultaneously. The patient’s prostheses needed to be fabricated in occlusion, but she lacked proper occlusion except for her second molar teeth (Figure 6).

**Figure 7:**
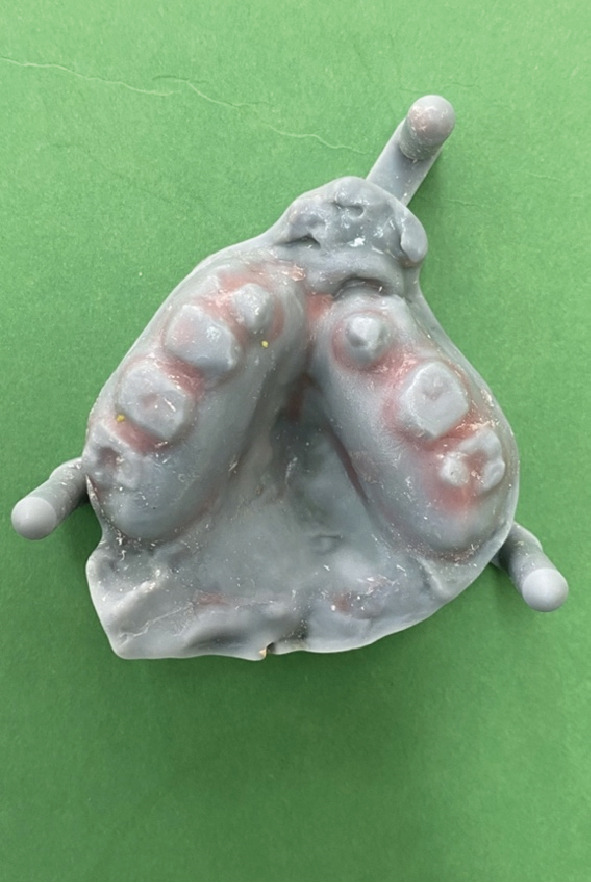
Printed cast with relief areas.

**Figure 8:**
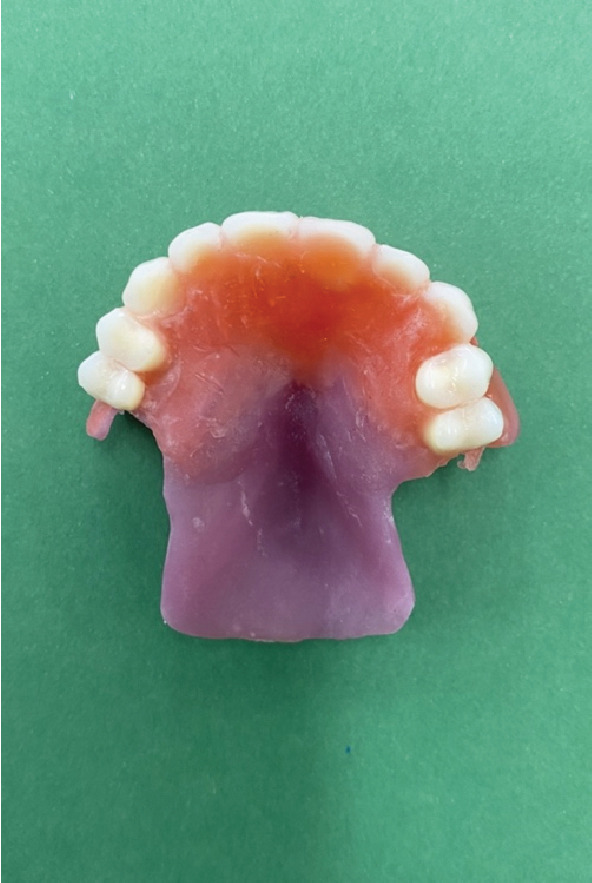
Acrylic base and teeth, arranged based on the occlusion.

**Figure 9:**
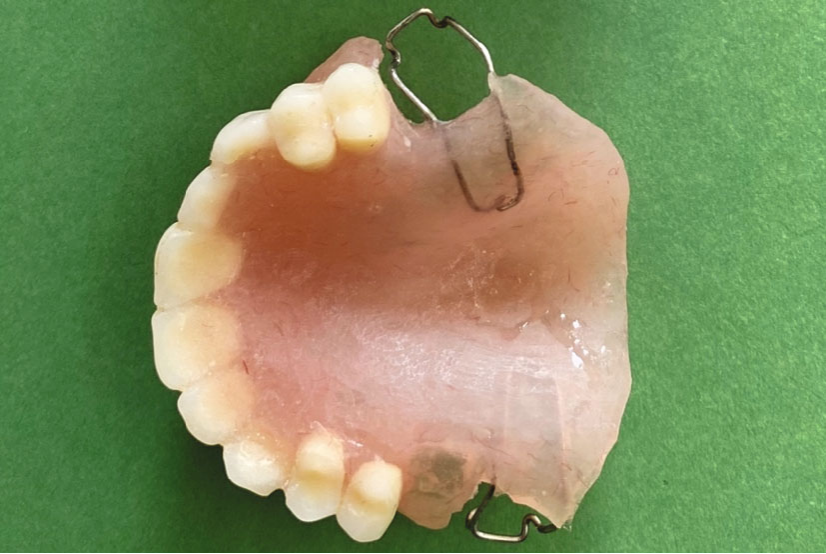
The final prosthesis with Adams clasp for retention.

**Figure 10:**
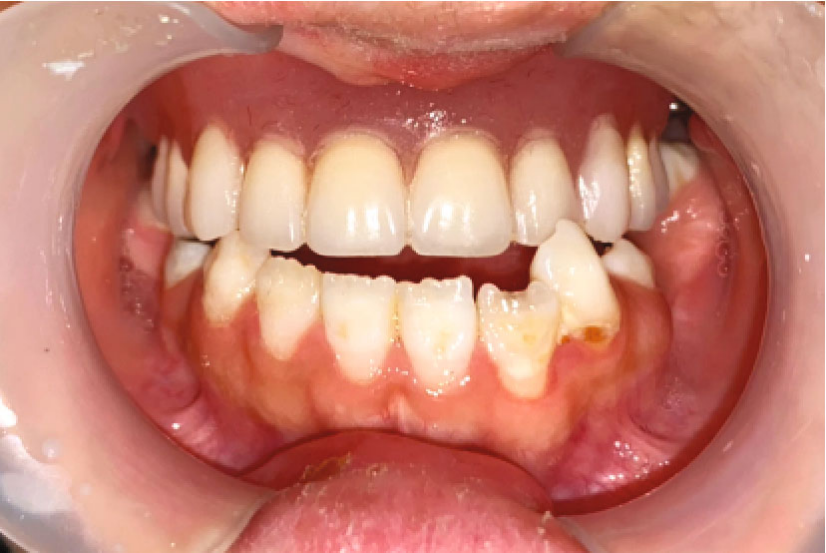
The final prosthesis in the mouth in centric relation position.

Relief areas were created for the undercut regions based on the printed casts, and the base was fabricated accordingly (see Figure 7). The teeth were arranged in accordance with the printed occlusion (Figure 8). Subsequently, the base, along with the teeth, was tried in the mouth, and the problem of lack of retention was addressed by the placement of Adams clasps (Figure 9) on the molars. After adjustments, the final prosthesis was delivered to the patient, and adjustments were made (Figures 1b and 10). The importance of attending follow‐up appointments and addressing any potential issues that may arise in the future was also thoroughly explained.

During follow‐up sessions, the patient reported a significant degree of satisfaction following the fitting of the prosthesis. They indicated that the restoration not only enhanced their aesthetic appearance but also contributed positively to their self‐esteem. Furthermore, the patient noted that the previously encountered issue with liquid consumption was effectively addressed through the use of the obturator.

## 3. Discussion

In managing young patients, especially those who are not accustomed to the dental clinical setting, conventional impression techniques can pose challenges, exacerbated by the aforementioned complications. As a result of discussions with the parents and after obtaining informed consent, the clinician decided to use digital technology for capturing maxillary and mandibular impressions [1, 21].

The adoption of digital impression methods proved to be swift, precise, and secure compared to conventional techniques in treating the presented cases [1, 21]. Similar findings have been reported in studies focusing on various applications such as fabricating preliminary casts, orthodontic devices, prosthetic rehabilitations, and even impression making for presurgical orthopedic appliances in neonates [1, 21]. While irreversible hydrocolloids like alginate can yield accurate oral structure records, they are difficult to control, potentially leading to displacement of the impression material into undesired areas such as the nasal cavity or pharynx [22–25]. Additionally, issues such as limited mouth opening and the difficulty of properly positioning the impression tray in the mouth, as well as the activation of the gag reflex, are disadvantages of this method.

Utilizing an intraoral scanner streamlines the impression process by eliminating the need for trays, impression materials, or casts in certain scenarios [21]. Moreover, it addresses technical concerns associated with standardizing mixing and setting times of conventional impression materials [21]. Although the initial investment in advanced digital equipment may be considerable, the resulting decrease in chair time and improved patient comfort can ultimately mitigate the financial expenditure over time [21].

Digital impressions offer enhanced accuracy by avoiding issues such as shrinkage, distortion, voids, or insufficient material, thus providing similar precision and improved reproducibility compared to conventional techniques for recording dental hard tissues [26, 27]. However, further investigation is needed to assess their effectiveness in capturing soft tissues and edentulous arches [21]. The use of intraoral scanners in situations where soft tissue exhibits significant mobility can lead to inaccuracies in the scanning process, consequently resulting in restorations with reduced fit. Nonetheless, in this particular case, the challenge was not encountered due to the positioning of the cleft in the palatal region and its lack of mobility.

In this patient, it was possible to utilize conventional methods for impression taking, and the accuracy of the work would likely have been comparable to that of the digital method. However, due to the patient’s small oral cavity and active gag reflex, obtaining the impression tray through conventional means proved challenging. Consequently, digital approach was chosen for the treatment.

Recent advancements have led to increased reproducibility in evaluating cleft care outcomes and enhanced self‐reported comfort and time efficiency with digital impressions compared to conventional methods [28]. In this case, the stability and retention of delivered appliances were satisfactory, with accuracy and reproducibility comparable to conventional impressions. Additionally, patients responded positively to intraoral scanner usage, with procedure times aligning with previous studies and being well‐received by children [15, 29]. Guardian presence during scanning was supportive, consistent with previous reports [28].

Follow‐up appointments were scheduled every 2 to 3 months to monitor appliance stability, with new intraoral scans and appliance fabrication if stability is compromised. Patients receive ongoing speech therapy until surgical fistula closure if necessary. Digital technology presents several advantages as an impression capture alternative, particularly beneficial for young patients with cleft lip and palate deformities. The patient did not return for a longer follow‐up due to satisfaction with the prosthesis and the fact that she was a student living far from the urban center. However, it is recommended that other patients undergo longer‐term follow‐up to assess the durability and long‐term success of obturator prostheses fabricated using digital methods.

## Consent

Considering the child being under 18 years old, which is below the legal age, permission was obtained from her parent to use her images in this journal.

## Conflicts of Interest

The authors declare no conflicts of interest.

## Author Contributions

Zahra Mohammadi: acquisition of data, analysis of data, drafting of article and/or critical revision, and final approval of manuscript. Sayed Shojaedin Shayegh: conception and design of the study, acquisition of data, drafting of article and/or critical revision, and final approval of the manuscript. Mohammad Amin Bafandeh: conception and design of the study, final approval of the manuscript, and analysis of data. Mohammad Reza Nazemalroaya: data gathering (make species and run the tests), analysis of data, and drafting of article.

## Funding

No funding was received for this manuscript.

## Data Availability

The data that support the findings of this study are available from the corresponding author upon reasonable request.
